# Prevention of ICU delirium and delirium-related outcome with haloperidol: a study protocol for a multicenter randomized controlled trial

**DOI:** 10.1186/1745-6215-14-400

**Published:** 2013-11-21

**Authors:** Mark van den Boogaard, Arjen J Slooter, Roger JM Brüggemann, Lisette Schoonhoven, Michael A Kuiper, Peter HJ van der Voort, Marga E Hoogendoorn, Albert Beishuizen, Jeroen A Schouten, Peter E Spronk, Saskia Houterman, Johannes G van der Hoeven, Peter Pickkers

**Affiliations:** 1 Department of Intensive Care Medicine, Radboud University Nijmegen Medical Centre, P.O. box 9101, internal post 710, 6500HB Nijmegen, Netherlands; 2 Department of Intensive Care Medicine, University Medical Centre Utrecht, PO box 85090, 3508AB Utrecht, Netherlands; 3 Department of Clinical Pharmacy, Radboud University Nijmegen Medical Centre, PO box 9101, 6500HB Nijmegen, Netherlands; 4 Scientific Institute for Quality of Healthcare, Radboud University Nijmegen Medical Centre, PO box 9101, 6500HB Nijmegen, Netherlands; 5 Department of Intensive Care Medicine, Medical Centre Leeuwarden, Henri Dunantweg 2, 8934AD Leeuwarden, Netherlands; 6 Department of Intensive Care Medicine, Onze Lieve Vrouwe Gasthuis, Oosterpark 9, 1091AC Amsterdam, Netherlands; 7 Research Department of Anesthesiology & Intensive Care, ISALA clinic, PO box 10400, 8000GK Zwolle, Netherlands; 8 Department of Intensive Care Medicine, Medical Spectrum Twente, PO box 50000, 7500KA Enschede, Netherlands; 9 Department of Intensive Care Medicine, Canisius Wilhelmina Hospital, PO box 9015, 6500GS Nijmegen, Netherlands; 10 Department of Intensive Care Medicine, Gelre Hospitals, Location Lukas, Albert Schweitzerlaan 31, 7334DZ Apeldoorn, Netherlands; 11 Catharina Hospital, Michelangelolaan 2, 5623EJ Eindhoven, Netherlands; 12 Department of Intensive Care Medicine, Radboud University Nijmegen Medical Centre, PO box 9101, 6500HB Nijmegen, Netherlands

**Keywords:** Critically ill, Delirium, Haloperidol, Intensive care, Mortality, Prevention, Prophylaxis, Randomized trial

## Abstract

**Background:**

Delirium is a frequent disorder in intensive care unit (ICU) patients with serious consequences. Therefore, preventive treatment for delirium may be beneficial. Worldwide, haloperidol is the first choice for pharmacological treatment of delirious patients. In daily clinical practice, a lower dose is sometimes used as prophylaxis. Some studies have shown the beneficial effects of prophylactic haloperidol on delirium incidence as well as on mortality, but evidence for effectiveness in ICU patients is limited. The primary objective of our study is to determine the effect of haloperidol prophylaxis on 28-day survival. Secondary objectives include the incidence of delirium and delirium-related outcome and the side effects of haloperidol prophylaxis.

**Methods:**

This will be a multicenter three-armed randomized, double-blind, placebo-controlled, prophylactic intervention study in critically ill patients. We will include consecutive non-neurological ICU patients, aged ≥18 years with an expected ICU length of stay >1 day. To be able to demonstrate a 15% increase in 28-day survival time with a power of 80% and alpha of 0.05 in both intervention groups, a total of 2,145 patients will be randomized; 715 in each group. The anticipated mortality rate in the placebo group is 12%. The intervention groups will receive prophylactic treatment with intravenous haloperidol 1 mg/q8h or 2 mg/q8h, and patients in the control group will receive placebo (sodium chloride 0.9%), both for a maximum period of 28-days. In patients who develop delirium, study medication will be stopped and patients will subsequently receive open label treatment with a higher (therapeutic) dose of haloperidol. We will use descriptive summary statistics as well as Cox proportional hazard regression analyses, adjusted for covariates.

**Discussion:**

This will be the first large-scale multicenter randomized controlled prevention study with haloperidol in ICU patients with a high risk of delirium, adequately powered to demonstrate an effect on 28-day survival.

**Trial registration:**

Clinicaltrials.gov: NCT01785290.

EudraCT number: 2012-004012-66.

## Background

Delirium is a neuropsychiatric disorder characterized by an acute onset of confusion, inattention, and a change in level of consciousness that tends to fluctuate during the day [1,2]. The incidence of delirium in intensive care unit (ICU) patients is high [3-6], approximately 30–50%. Further, its occurrence is associated with detrimental outcome, including prolonged duration of mechanical ventilation, increased ICU and hospital length of stay (LOS) [4-6], unplanned removal of tubes and catheters [6], increased mortality [6-8], and long-term cognitive disturbances [9,10]. Therefore, a strategy that can prevent delirium in high-risk patients may also decrease short-term (i.e., 28-day) mortality.

The first step in the management of delirium is to treat the underlying disease. If delirium does not subside, haloperidol is the most frequently used pharmacologic treatment of delirium. However, this drug may also be used to prevent delirium. In non-ICU patients, beneficial effects have been reported of prophylactic haloperidol in older and surgical patients [11,12]. For critically ill patients, data concerning prevention with anti-psychotic drugs are scarce and inconsistent [13-15]. In one retrospective cohort study, ICU patients who received haloperidol were found to have a lower mortality rate compared to ICU patients that did not receive haloperidol [16]. Only one prospective, randomized trial in critically ill patients is available, showing that haloperidol prophylaxis in non-cardiac surgical ICU patients had beneficial effects on delirium incidence and the number of delirium free-days [17]. However, only a very low dose, for 12 h infusion of haloperidol was administered and many of the patients were in the ICU for less than 24 h [17]. Thus, there are no large, definitive trials that have studied the effect of haloperidol prophylaxis in ICU patients.

Recently, a model was developed and validated to predict delirium in ICU patients [18]. With this model, the effectiveness of preventive strategies can be compared in different groups that differ in *a priori* risk of delirium. This delirium prediction model was used to identify high-risk ICU patients (i.e., *a priori* risk of delirium >50%). Compared to a historical control group, prophylactic low-dose haloperidol was associated with better delirium related outcomes. In this high-risk group (>50%) of critically ill patients, incidence of delirium was significantly lower in the preventive treatment group (65%), compared to the control group (75%). Furthermore, haloperidol prophylaxis was associated with a hazard rate of 0.8 for 28-day mortality [19]. However, because of the pre-/post-implementation design of this trial, other factors that may have changed between the study periods may have influenced these findings. Nevertheless, the results suggest that prevention of delirium might be beneficial in critically ill patients. Since no relevant side effects associated with the administration of a low dosage of haloperidol were reported in the prophylactic studies [11,12,17,19], it is conceivable that a higher dose of haloperidol may exert more pronounced beneficial effects. Furthermore, the optimal dosage of haloperidol in critically ill patients, as well as the occupancy levels of dopamine-2 receptors and sigma-1 receptors when administering haloperidol, remain unknown. Therefore, in the present study proposal the effects of two dosages of haloperidol versus placebo will be determined.

Given the fact that the development of delirium can be predicted only 24 h after ICU admission, and the clear relation between time to treatment with haloperidol and the duration of delirium [19], initiation of prophylaxis as early as possible is warranted. Based on our historical data, the median predicted risk of delirium is approximately 35% in patients with an expected stay on the ICU of over one day; this is considered a high risk for delirium development. A clinical estimation of an ICU LOS >1 day is possible within the first few hours after ICU admission. Previous studies that included patients using similar criterion of an expected ICU LOS were found feasible [20,21]. Therefore, the clinical estimation by the attending ICU-physician of an ICU LOS >1 day will be used as an inclusion criterion for this study, in which the prediction to develop delirium (using the PRE-DELIRIC model [18]) will be used to facilitate post-hoc analyses to investigate which patients benefit most from prophylactic therapy. By using the PRE-DELIRIC score as an *a priori* determined post-hoc analysis, we will learn if the putative beneficial effect of haloperidol is related to the risk to develop delirium, or comparable in all patients, independent of the baseline risk to develop delirium. In addition, it will facilitate the determination of a cut-off level for the PRE-DELIRIC score.

Potential side effects of haloperidol include extra pyramidal symptoms, drowsiness, agitation, and ventricular arrhythmias. The latter are extremely rare (only case-reports are published [22-25]) and dose dependent. With the haloperidol dosage that will be used in the present study (3x1 mg or 3x2 mg intravenously daily), no relevant side effects are expected, regardless of underlying condition, organ dysfunction, and concomitant medication. Nevertheless, and given the preventive nature of this study, extra attention is being paid to the recognition of possible side effects of haloperidol that could mitigate its potential beneficial effects. Importantly, in three recent prophylactic haloperidol studies [11,17,19] no relevant side effects, and in particular no ventricular arrhythmias, were reported using similar low dosages of haloperidol as described in the present protocol. Regarding the choice of haloperidol dosage, this was based on the previous study in which no relevant side effects were found when using a prophylactic dose of 3x1 mg in delirium high-risk ICU patients, and resulted in a significant decrease of delirium incidence [19]. However, in this high-risk group (mean delirium risk of 75%) the incidence was still 65%, suggesting that a higher dosage of haloperidol might decrease the incidence of delirium to a greater extent. For this reason, and taking into account the non-relevant side effects, we choose to add a third arm using a higher dose of 3x2 mg haloperidol.

The aim of this study is firstly to determine the effect of two different low dosages of prophylactic haloperidol on 28-day mortality as well as the incidence of delirium and other delirium related outcome measures and quality of life, compared with placebo, in ICU patients with an expected ICU stay of >1 day. Secondly, to relate the potential beneficial effects of haloperidol to the *a priori* risk of developing delirium, using the PRE-DELIRIC model [18]. Lastly, to evaluate possible side effects of prophylactic haloperidol.

## Methods

This study protocol is in accordance with the CONSORT guidelines concerning randomized controlled trials [26].

### Design and setting

A prospective, multicenter, three-armed, permuted block-randomized, double-blind, placebo-controlled, prophylactic intervention study will be conducted in critically ill patients with a high risk of delirium development. All participating sites are members of the Dutch Intensive Care Unit Delirium Consortium facilitating the collaboration related to delirium research in the ICU. In all centers, patients are screened for delirium using the validated Dutch translation of the Confusion Assessment Method-Intensive Care Unit (CAM-ICU) [27-29] by well-trained ICU nurses, at least two times a day and more often if indicated (during periods of fluctuating symptoms or levels of sedation). Before initiation of the study, ICU nurses will receive additional information concerning the CAM-ICU on top of their in-house CAM-ICU training. Furthermore, during the study, inter-rater reliability measurements of the CAM-ICU and the Richmond Agitation Sedation Score (RASS), as part of the CAM-ICU, will be performed in order to check the quality of the assessments, and if necessary, additional training will be provided.

The study will start after training of all centers including an initiation visit.

### Study population

#### ***Inclusion criteria***

All consecutive critically ill patients admitted to the ICU aged ≥18 years at the time of ICU admission and with an expected length of ICU stay of over one day will be included as informed consent is obtained.

Data from patients that are included in the study, but who do not receive any study drug and are discharged from the ICU within 24 h will be discarded from further analysis. Patients who received at least one dose will remain in the study and patients in whom study medication had to be halved or stopped, e.g., because of prolonged QTc time or other side effects, will remain allocated to their study group and analyzed on an intention-to-treat basis.

#### ***Exclusion criteria***

The following will be considered exclusion criteria: no informed consent obtained; history of epilepsy, Parkinson’s disease, hypokinetic rigid syndrome, dementia or alcohol withdrawal syndrome; patients admitted to the ICU for any other neurological disease (including post-cardio-pulmonary resuscitation patients, and patients admitted with coma due to overdose); patients treated with anti-psychotic therapy over the last 30 days prior to ICU admission; prolonged QTc-time (>500 msec) or history of clinically relevant ventricular arrhythmia in last 12 months; pregnancy/breast feeding; documented delirium prior to ICU admission; reasons that impair delirium assessment with the CAM-ICU (serious auditory or visual disorders, unable to understand Dutch or English; severely mentally disabled; serious receptive aphasia); ICU-stay less than one day; moribund and not expected to survive two days; known allergy to haloperidol.

### Objectives

Definitions of variables in objectives are reported in Table 1.

**Table 1:** Definition of study objectives

**Objective**	**Definition**
*Survival days in 28 days*	Number of days that patients survive in 28 days. All patients will be classified as either ‘alive at study day 28’ or, if dead, ‘dead at study day 28’ on an intention-to-treat basis.
*Delirium diagnosis*	Patients are diagnosed as delirious when they have at least one positive CAM-ICU screening during their complete ICU stay. Patients who were not delirious during their ICU-stay are considered as non-delirious patients.
*Delirium-and-coma-free days in 28 days*	Number of days that the patient is alive and not delirious and not in coma over 28 days starting from the day of inclusion. A delirium-and-coma-free day is defined as a negative CAM-ICU screening with a Richmond Agitation Sedation Score (RASS) greater than (more alert than) -3/-4/-5 during a day. In case a delirious patient is discharged to the ward, a delirium-free day is defined as a delirium observation scale score [30] of less than 3 during a complete day.
*Duration of mechanical ventilation*	Time in days that the patient is on the mechanical ventilator. If the patient is ventilated mechanically several times during one ICU admission, then the ventilator times are added. Both invasive and non-invasive ventilation will be registered. Ventilator-free days (in 28 days) will be calculated.
*Incidence of re-intubation*	Patients who need to be intubated within 28 days from randomization, following a previous extubation, irrespective of the reason for re-intubation, are counted as incident case for re-intubation.
*Incidence of ICU readmission*	Patients who need to be readmitted to the ICU during within 28 days from randomization, irrespective of the reason for readmission, are counted as incident cases for ICU readmission.
*Side effects*	Drowsiness, agitation, QTc-time prolongation (using 12-leads ECG or monitor strip with Bazett’s formula) and development of extra pyramidal symptoms such as tandem gait, dystonia, tremor, myoclonus, tics, rigidity, akathisia [31], determined daily by physical examination by the intensivist.
*Serious adverse event*	Any untoward medical occurrence or effect at any dose that results in one of the following outcomes and is not classified as a clinical outcome of delirium or the underlying disease using the description above:
	- death that is not related to the underlying disease or sequel of the underlying disease, or death that is considered by the investigator to be related to study drug
	- prolonged inpatient hospitalization or re-hospitalization
	- a life-threatening experience (that is, immediate risk of dying)
	- persistent or significant disability/incapacity
	- congenital anomaly/birth defect
	- considered significant by the investigator for any other reason
*Sudden unexpected serious adverse reactions*	Unexpected adverse reactions are adverse reactions, of which the nature, or severity, is not consistent with the applicable product information. Adverse reactions are all untoward and unintended responses to an investigational product related to any dose administered.

#### ***Primary objective***

To determine the effect of prophylactic haloperidol use on 28-days survival.

#### ***Secondary objectives***

There are seven secondary objectives:

1. To determine the effect of prophylactic haloperidol use on 90-day survival; survival analysis stratifying for delirium incidence will be performed.

2. To determine the effect of prophylactic haloperidol use on the incidence of delirium.

3. To determine the effect of prophylactic haloperidol use on the number of delirium- and coma-free days in a period of 28 days.

4. To determine the effect of prophylactic haloperidol use on delirium related outcomes: time to successful extubation, incidence of re-intubation, incidence of ICU readmission, and incidence of unplanned removal of tubes and catheters.

5. To evaluate the incidence and severity of side effects of haloperidol prophylaxis.

6. To determine the effect of preventive haloperidol use on quality of life 1 and 6 months following ICU admission compared with a baseline measurement at ICU admission using the Short Form-12 (SF-12) questionnaire.

7. *A priori* defined post-hoc analyses:

a. To determine the preventive effectiveness of haloperidol in different patient groups based on the *a priori* risk to develop delirium: patients with a predicted risk up to 50%, 50–70%, 70–90%, above 90% will be evaluated.

a. To determine the preventive effectiveness of haloperidol in different patient groups: medical/surgical/trauma patients, per acute physiology and chronic health evaluation-II (APACHE-II) score (<20, 20–25, >25)

a. Effectiveness of prophylaxis when delirium is diagnosed based on 1, 2, or more days with positive CAM-ICU scoring.

### Randomization

Block randomization will be applied by the pharmacist. The randomization numbers are coupled with the Clinical Report Form (CRF) number and the number of study medication box. Boxes are numbered and consist of 12 ampoules of study drug. If necessary, when a patient is admitted to the ICU for more than 4 days and is not delirious, a new box will be assigned to this patient consisting of the same study regime as the previous box. All study medication will be manufactured by the Department of Clinical Pharmacy of the Radboud University Nijmegen Medical Centre, which holds a Good Manufacturing Practice certificate. Ampoules of study medication consist of 1 mg/mL or 2 mg/mL haloperidol or 1 mL sodium chloride 0.9% solution, all with a total volume of 1 mL. The randomization code is kept by the pharmacist and will be broken only if necessary for safety reasons. Only the pharmacist of the Radboud University Nijmegen Medical Centre is unblinded for this study.

### Intervention and control group

This study involves a comparison of prophylactic haloperidol in a dosage of 1 mg (intervention group 1) or 2 mg (intervention group 2) administered as a bolus intravenously three times a day compared with placebo of 0.9% sodium chloride (control group) administered intravenously three times a day in a double-blind fashion. All ampoules and drug boxes have an identical appearance.

To decrease the likelihood of encountering side effects in specific cohorts, the dose of the study drug will be halved in patients aged ≥80 years, weighing ≤50 kg, suffering from liver failure (indicated by serum bilirubin level >50 μmol/L) present at time of inclusion or during the study. Patients with an adjusted dose remain allocated to their original group (intention-to-treat).

Patients with an adjusted dose remain allocated to their original group (intention to treat).

### End of study medication

Prophylactic treatment with haloperidol will be continued until day 28, or discharge from the ICU (whatever comes first), or until delirium occurs. In the latter case, patients will be subsequently treated according to the delirium treatment protocol (with higher dosage of open label haloperidol).

In case delirium occurs, the patient’s study medication (prophylaxis treatment) will be stopped and the patient will be treated with open label haloperidol according to the delirium treatment protocol described below. Analysis will be on intention-to-treat basis. Study medication will not be restarted once delirium subsides, therapeutic haloperidol is stopped, or when a patient is re-admitted to the ICU within 28 days.

#### ***Delirium treatment***

Patients with delirium (defined as a positive CAM-ICU test) will be treated with 2 mg haloperidol intravenously three times daily. Patients suffering from *hypoactive* delirium (only RASS scores between 0 and -3) and patients aged ≥80 years, weighing ≤50 kg, or with liver failure, will be treated with a lower dosage of 3x1 mg intravenously. Dosage can be increased up to a maximum of 3x5 mg in case of serious agitation or anxiety due to delirium. Additionally, midazolam, clonidine, propofol, or dexmedetomidine can be used as an escape therapy in case of serious agitation with insufficient efficacy of haloperidol according to usual patient care.

In patients treated for more than three days, the dosage will be halved when delirium subsides. When delirium does not reoccur the following day (CAM-ICU remains negative), the dosage will be halved again and then stopped the third day when the patient remains non-delirious. In case delirium occurs again during the reducing phase, the original dose will be restarted.

### Data collection

All data will be collected electronically in an electronic CRF (E-CRF). The E-CRF is a secured website where the participating hospitals must login using a unique password. Participating hospitals will only have access to their own data.

Demographic variables will be collected from all patients, including age, gender, PRE-DELIRIC score, APACHE-II score, and diagnosis group. Furthermore, outcome related variables will be collected (Table 2) as well as variables that may influence the delirium outcome, i.e., dexmedetomidine [32] and early mobilization [33]. Concerning the study medication, we will collect data on the amount of study drug administered as well as open-label haloperidol and other anti-delirium drugs administered in case delirium occurs.

**Table 2:** Primary, secondary endpoints and posthoc analyses

**Primary endpoint**	**Secondary endpoints**	**Post-hoc analyses**
28-day mortality	90-day mortality Delirium incidence	Effectiveness in groups of predicted risk up to 50%, 50–70%, 70–90%, above 90% will be evaluated
	Number of delirium and coma free-days in 28 days	Effectiveness in diagnosis groups: medical, surgical and trauma patients
	Duration of mechanical ventilation	Effectiveness per APACHE-II score: <20, 20–25, >25
	Incidence of unintended tube or catheter removal	Effectiveness in groups with 1, 2, or more positive CAM-ICU scores
	Incidence of ICU re-admission	
	Quality of life measured at time of ICU admission, after 1 and 6 months using SF-12	
	Incidence and severity of side effects of prophylactic haloperidol (all patients)	

### Safety monitoring and safety issues

A Data Safety Monitor Board (DSMB) is established for this study to perform ongoing safety surveillance and to perform interim analyses on the safety data. This is an independent committee composed of two physicians/researchers (a psychiatrist and an anesthetist) and a statistician. The DSMB will perform all interim analyses. To assess side effects of haloperidol and to determine superiority of the intervention or placebo the DSMB will be unblinded for this study, while all researchers remain blinded. Furthermore, the study will be monitored by an independent researcher who will monitor the trial master file, and perform random checks of informed consent forms, drug accountability lists, and source data of primary and secondary outcome measures.

Concerning safety issues, during a patient’s treatment with the study medication, known side effects of haloperidol [34] are pro-actively collected with a special focus on prolonged QTc-time, drowsiness, extrapyramidal symptoms, and agitation and sedation effects. All side effects will be collected during patients’ treatment with the study medication until 24 h after stopping the study medication. In case of the occurrence of side effects, physicians can reduce the dosage or stop the study medication, depending on the severity of the occurred side effect and at the discretion of the attending physician. Only for prolonged QTc-time, strict stopping rules are applied, as described below. To detect side effects of haloperidol, the protocol dictates that patients are physically examined every day for known signs of extrapyramidal symptoms (signs of Parkinsonism and/or akathisia and/or dystonia). Furthermore, daily QTc-time is calculated using a 12-lead ECG or a monitor lead ECG. A QTc-time of over 500 msec combined with an increase of over 10% of baseline QTc-time is defined as prolonged QTc-time. In case of QTc-time prolongation, the study drug is temporarily stopped until QTc-time is normalized. After normalization of QTc-time (<500 msec) the study drug will be restarted. If QTc-time becomes prolonged again, the study drug will be stopped definitively. The patient will remain allocated to the original study group.

To determine long-term side effects, such as rigidity, and quality of life, a recommended and validated quality of life questionnaire, SF-12 [35], is taken at the time of admission and is sent to the patients 1 and 6 months after ICU admission.

The following adverse events will be collected: serious adverse events; non-serious adverse events that are considered by the investigator to be possibly related to the study drug (e.g., prolonged QTc-time, drowsiness, extrapyramidal symptoms, agitation and sedation effects); adverse events that lead to permanent discontinuation of the study drug administration. A serious adverse event is any untoward medical occurrence or effect at any dose that results in one of the following outcomes and is not classified as a clinical outcome of delirium or the underlying disease using the description: death that is not related to the underlying disease or sequel of the underlying disease, or death that is considered by the investigator to be related to the study drug; prolonged inpatient hospitalization or re-hospitalization; a life-threatening experience (that is, immediate risk of dying); persistent or significant disability/incapacity; considered significant by the investigator for any other reason.

### Sample size calculation and statistics

**Figure 1:**
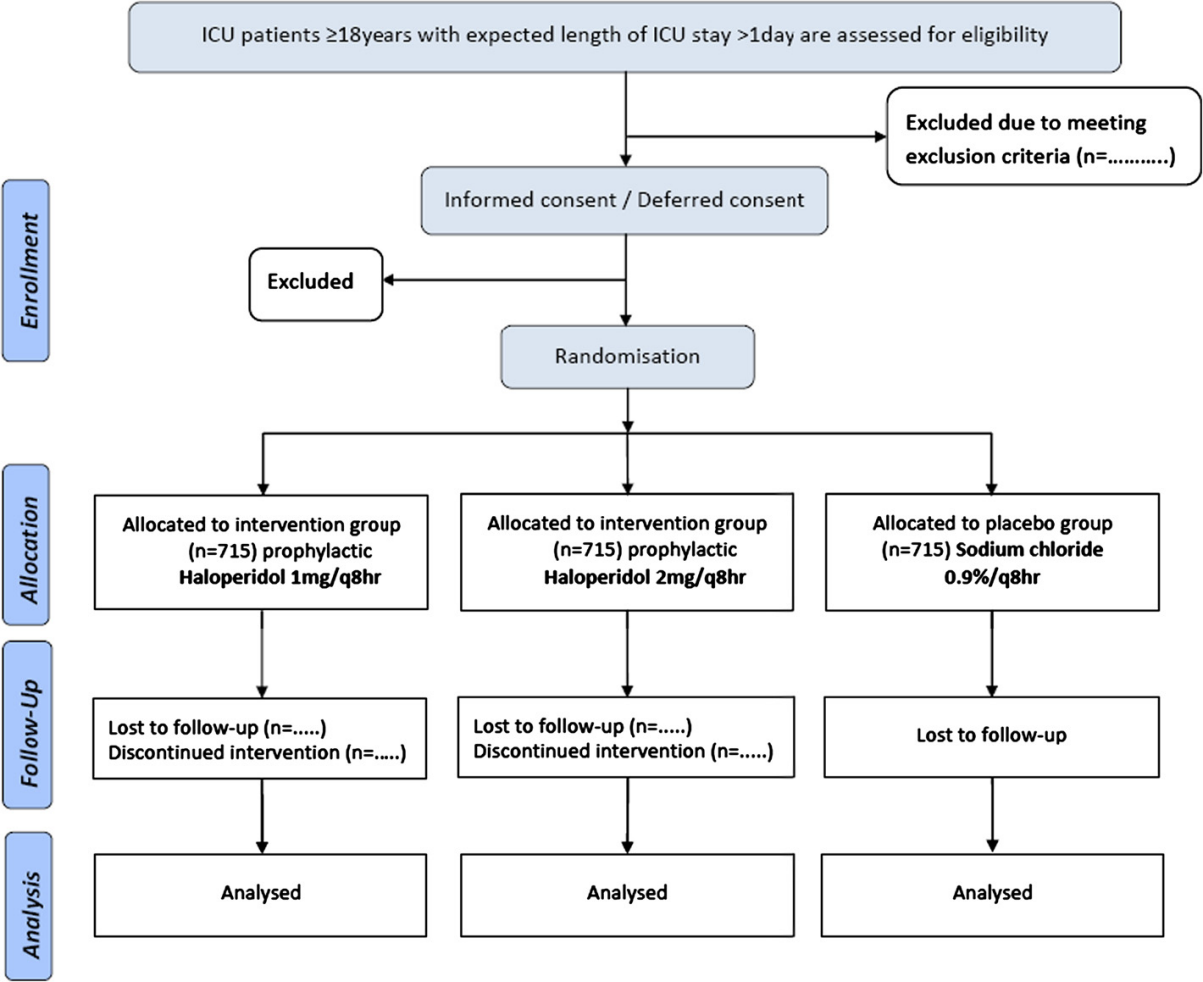
CONSORT flowchart of patient enrollment.

Sample size calculation is based on the difference in survival from our previous prophylactic haloperidol study [19]. In this study, the median survival time in the control group was 18 days. If the true hazard ratio of control patients relative to intervention patients is 0.85, taking into account an accrual time of 90 days with 28 days of follow-up, we will need to study 647 patients per intervention group and 647 control patients to be able to reject the null hypothesis that the experimental and control survival curves are equal with probability (power) 0.80. The Type I error probability associated with this null hypothesis test is 0.05. Taking into account a dropout percentage of 10%, we will include 715 patients per group (Figure 1). A fixed sequence procedure will be followed. First, the highest dosage of prophylactic haloperidol will be compared with placebo using alpha 0.05 (two-sided). Only if H_0_ is rejected, subsequently the lower dosage of prophylactic haloperidol will be compared with placebo. Cox regression analysis will be used to test differences on 28-day survival in the intervention group compared with the placebo group.

A safety, futility, and superiority interim analysis will be performed by the DSMB after 175, 350, 500 (safety and futility) and 1,000 (safety and superiority) patients are included. Differences in serious adverse events between intervention and placebo are used for safety analyses and the primary endpoint 28-day survival is used to determine futility or superiority of intervention or placebo.

After the inclusion of 1,000 patients, superiority will be tested. A proven superiority (*P < 0.003, two-sided)* of any dose of haloperidol over placebo or a proven superiority (*P < 0.003, two-sided)* of placebo over haloperidol determined during the interim analysis will result in an alpha of 0.049 (two-sided) for the final analysis. This alpha distribution was calculated by an independent statistician according to the method of Lan-DeMets cumulative alpha spending function of O’Brien-Fleming alpha spending [36].

Data will be analyzed according to the intention-to-treat principle. For the descriptive statistics, continuous variables will be given as mean with standard deviation or median and inter quartile ranges, depending on their distribution. Normally distributed variables will be tested using Student’s *t*-test for comparison and Mann-Whitney U-tests for non-normally distributed variables. Categorical (and binary) variables will be presented as numbers and percentages and will be analyzed using the χ^2^ test. Survival analyses with Kaplan-Meier curves will be used as graphical representation. Cox proportional hazard regression analyses will be used to estimate the hazard ratio for survival with the use of haloperidol versus placebo. Furthermore, we will perform adjusted analyses for relevant covariates (including delirium, APACHE-II score, age, sex, sepsis, and other differences between the three study groups irrespective of the possible imbalance between the three groups). For this covariate analysis, univariate logistic regression analysis will be performed first in order to test the strength of the relationship. Subsequently, variables significantly associated with the dependent variable will be included in the covariate analyses.

Furthermore, subgroup analysis will be performed by direct comparisons of *a priori* specified subgroups, i.e., predicted risk group, admission type group, and APACHE-II group, in order to determine effectiveness of prophylactic haloperidol treatment in these subgroups on 28-day survival.

All statistical tests are two-sided and statistical significance is defined as a *P* value < 0.05. All data will be analyzed using SPSS version 20.01 (SPSS, Chicago, IL, USA).

### Ethics

The study will be conducted according to the principles of the Declaration of Helsinki (version 2008) and in accordance with the Medical Research Involving Human Subjects Act.

There are indications that early treatment of delirium is more effective than delayed treatment [37], but this needs to be confirmed in other studies. Since it is recognized that the onset of delirium occurs, on average, on day 2 after ICU admission, early preventive treatment is likely to be more effective. In the short-term, low dose haloperidol has no known relevant side effects, therefore randomization and administration of study medication will be started immediately following identification of a high-risk patient. When an informed consent procedure results in a delay of randomization, a deferred consent procedure may be followed. The informed consent procedure will be started as soon as possible, but always within 24 h following ICU admission. If no deferred informed consent can be obtained within 24 h, the patient will be excluded from the study and study drug administration will be stopped.

The medical ethical committee of Arnhem-Nijmegen (CMO) approved this study including this deferred consent procedure (CMO-number 2012/424). This trial is registered on clinicaltrials.gov: NCT01785290.

## Discussion and trial status

This is the first large-scale multicenter randomized-controlled prevention study with haloperidol in ICU patients with an increased risk to develop delirium. The study design and protocol was finalized in 2012 and the protocol passed the medical ethical committee on February 2013. Prior to the start of the study, all centers will be visited to inform all involved researchers and a second meeting will take place as an initiation visit. Furthermore, a meeting with the DSMB and the monitor will be scheduled prior to the conduction of the study.

Results of our study will be of importance for critically ill patients and permits us to draw conclusions on the effectiveness of haloperidol prophylaxis in reducing 28-day mortality, preventing delirium, and improving delirium-related outcome. Furthermore, it will provide information regarding the delirium-risk category in which the PRE-DELIRIC model prophylaxis is most effective. Positive effects of haloperidol prophylaxis on delirium outcome will result in the alteration of daily practice for critically ill patients and would therefore have major implications. However, if there is no effect of haloperidol prophylaxis, other possibilities of delirium prevention, such as nursing interventions, need to be studied. In addition, the effectiveness of nursing interventions focusing on delirium prevention can be studied in conjunction with haloperidol prophylaxis.

## Trial status

Preparations of the study are nearing completion. We expect that the study will start mid-2013.

## Abbreviations

APACHE-II: Acute physiology and chronic health evaluation-II; CAM-ICU: Confusion assessment method-intensive care unit; DSMB: Data safety monitoring board; E-CRF: Electronic clinical report form; ICU: Intensive care unit; LOS: Length of stay; RASS: Richmond agitation sedation scale.

## Competing interests

The authors declare that they have no competing interests related to this study.

## Authors’ contributions

MvdB, AS, and PP designed the study and wrote the protocol. MvdB is the principal investigator. PP is project leader and AS and JH are sub-investigators. AS, PP, and LS, will supervise the conduct of the study. SH supported the study design and protocol with statistical advice. RB supported the study protocol with regard to study drug advises. MvdB, AS, MK, PvdV, MH, AB, JS, and PS are responsible for conducting the study in their hospital. JvdH co-supervised and corrected the manuscript. All authors read and approved the final manuscript.
